# Renal arteriography with endovascular ultrasound for the management of renal infarction patients

**DOI:** 10.1186/s12882-020-01929-z

**Published:** 2020-07-14

**Authors:** Fabrice Ivanes, Jean Dewaele, Caroline Touboul, Philippe Gatault, Bénédicte Sautenet, Christelle Barbet, Matthias Büchler, Laurent Quilliet, Denis Angoulvant, Jean-Michel Halimi

**Affiliations:** 1 Department of Cardiology, CHRU Tours, Tours, France; 2 EA 4245 T2I & Loire Valley Cardiovascular Collaboration, Université de Tours, Tours, France; 3 Department of Nephrology, CHRU de Tours, Tours, France

**Keywords:** Renal infarction, Renal arteriography, Intravascular ultrasound, Secondary prevention

## Abstract

**Background:**

Renal infarction (RI) is a rare disease with poor prognosis. Appropriate secondary prevention treatment is essential and requires an exhaustive etiological assessment. We aimed to determine whether invasive endovascular explorations may improve the diagnostic process and change the secondary prevention treatment strategy in RI patients.

**Methods:**

We report a retrospective observational study of 25 RI patients referred to Tours University Hospital between 2011 and 2018 for etiological investigation including renal arteriography and intravascular ultrasonography (IVUS). We sought for antithrombotic treatment regimen, vital status, bleeding and ischemic outcomes during the median follow-up of 59 months.

**Results:**

Invasive explorations showed local arterial disease in 14 patients (56%). This led to a diagnosis or change in diagnosis in 9 patients (36%) and to a change in antithrombotic strategy in 56% of cases, with an increased prescription of antiplatelet therapy. No patient died, only two patients (8%) had persistent mild renal insufficiency. One IVUS complication was reported and treated without any significant long-term consequences.

**Conclusion:**

Invasive endovascular investigations of RI may modify the secondary prevention treatment through a better assessment of the aetiology of RI. Multicentric randomized studies are necessary to advocate the hypothesis that invasive exploration of renal artery can improve long-term prognosis.

## Background

Renal infarction (RI) is a rare disease that can be of multiple causes. It may result from an embolic mechanism, or from an abnormality of the renal arterial wall.

To date, there is no guideline regarding the antithrombotic treatment of RI either at the early phase or at long-term. The lack of guideline is an issue, considering the high recurrence of cardiovascular events and the poor prognosis of the disease. In retrospective studies, patients with RI have an all-cause mortality rate of 19.7% at 40 months [1], despite a rare evolution to dialysis or end-stage renal disease (2% at 41 months [2]). This means that the poor prognosis is not due to the severity of the kidney injury, but mainly to cardiovascular events and patients’ comorbidities with an outcome rate of 12% at 48 months [3], highlighting the need of an appropriate secondary prevention treatment. Following the same logic as in cerebrovascular accident, anticoagulant treatment is generally given in case of suspected embolic mechanism. On the contrary, antiplatelet therapy is preferred in case of arterial disease.

The classic investigations for renal artery abnormalities are however poorly informative. Renal artery doppler can only assess the presence and severity of a renal artery stenosis [4], and thus is not appropriate to detect non severe atherosclerosis and to identify the etiological mechanism of a narrowing. Enhanced computed tomography scan or MRI are useful to diagnose renal artery stenosis but are also insufficient to detect non-severe atherosclerotic plaque. Knowing this, an exhaustive etiological assessment of RI might include an invasive exploration of renal arteries. Angiography is considered the gold standard procedure for the diagnosis of artery disease, but it only visualizes the lumen of vessels, while atherosclerosis is a disease of the arterial wall. Atheroma may then be underdiagnosed despite several angiographic views. Unlike angiography, intravascular ultrasonography (IVUS) can directly assess atherosclerosis and the remodelling of arterial wall. For instance, coronary IVUS in very young cardiac transplant donors (less than 20 years old) showed atherosclerosis in 17% of cases, with an apparently normal coronary angiography [5]. Hence, IVUS is more sensitive than angiography for detecting vascular lesions, provided that the diseased artery is accessible to invasive catheterization.

The purpose of the present proof-of-concept study was to investigate whether renal artery invasive exploration could improve the diagnostic process and change the secondary prevention treatment strategy in a monocentric RI cohort.

## Methods

### Study population

This was a retrospective cohort study, including all patients diagnosed with RI of unknown origin and referred to the Cardiology Department of Tours University hospital for etiological investigations. Renal infarction was considered of unknown origin if the patient had no signs (or history) of atrial dysrhythmia or overt suspicion of intra cardiac thrombus in the initial non-invasive investigations. These patients had an invasive exploration between November 2011 and August 2018. Following routine procedure in our hospital, written informed consent was given by each patient before any invasive exploration. The study was approved by the institutional review board of the Pole Coeur Thorax Vaisseaux from the Trousseau Hospital (Tours, France) on December 2018 and was registered as a clinical audit. Participating patients’ non-opposition was recorded.

### Baseline characteristics

Data regarding the baseline demographic included: age, body mass index, history of diabetes, dyslipidaemia, high blood pressure, smoking habits, family history of cardiovascular disease, time from the onset of symptoms to invasive exploration. In addition, the pain characteristics, urine dipstick abnormalities, serum creatinine level and estimated glomerular filtration rate (through MDRD formula), white blood cell count, LDH, CRP, computed tomography scan report and involvement of one or both kidneys were included as baseline descriptors, as well as the delay between symptoms onset and RI diagnosis.

### Clinical and paraclinical evaluation

All patients were diagnosed with RI by contrast-enhanced computed tomography scan, in our university hospital or most commonly in a peripheral hospital. The initial symptom at presentation was mainly acute flank or abdominal pain. A renal artery abnormality, e.g. arterial dissection or narrowing, was sought in every scan report. The standard etiological exploration included 12-lead rest ECG and ECG Holter monitoring searching for sustained atrial arrhythmia, transthoracic echocardiography (TTE), transoesophageal echocardiography (TOE), and peripheral arterial doppler ultrasonography. Patients were also investigated for connective tissue (in case of clinical suspicion) or thrombophilia disorders.

### Renal artery angiography and IVUS

In order to perform an exhaustive search for arterial abnormalities, all patients underwent renal angiography. Following selective catheterization, a guide wire was consecutively inserted in both renal arteries, allowing the realization of IVUS in the main renal arteries and at least one of their side branches. The arterial branch responsible for RI was not always explored because of either total occlusion or unfavourable anatomy. Two different IVUS devices were used: ATLANTIS SR PRO 40 (BOSTON SCIENTIFIC) before 2012, and VOLCANO EAGLE EYE (VOLCANO) from 2012 and after. A motorized pullback device was used for both devices.

### Secondary prevention treatment

Considering standard and invasive explorations, the secondary prevention treatment was discussed. Following the same logic as in ischemic stroke secondary prevention, in the absence of a truly identified cardioembolic origin and in the presence of renal artery abnormalities (e.g. atherosclerosis, dissection and fibro dysplasia), patients were discharged with a prescription of antiplatelet agent, while the presence of an intra cardiac thrombus and/or sustained atrial dysrhythmia led to the prescription of an oral anticoagulant [6].

### Outcomes

Clinical and biological outcomes were gathered through medical reports and phone contact in January 2019: current antithrombotic treatment, RI recurrence, Major Adverse Cardiovascular and Cerebrovascular Events (MACCE) outcomes defined as 1) ischemic stroke or transient ischemic attack; 2) acute myocardial infarction; 3) acute decompensated heart failure; or 4) cardiovascular death. Bleeding events according to the Bleeding Academic Research Consortium (BARC [7]) classification were also collected, as well as a creatinine level and Glomerular filtration rate according to the MDRD (Modification of Diet in Renal Disease) formula. A new blood test was performed if the last creatinine measurement was older than 3 months.

### Data analysis

All quantitative variables were expressed as medians, with interquartile ranges, defined by the range between the first and the third quartile because of the small number of patients and the potential extreme values.

## Results

### Demographics and clinical baseline characteristics

25 patients were included in this study, whose baseline characteristics are presented in Table 1. The mean age was 47 years (interquartile range: 11) and 88% were male. There was a high prevalence of high blood pressure (52%), dyslipidaemia (32%) and current smoking (48%). Diabetes mellitus and family history of cardiovascular disease rates were low (8 and 16% respectively).

**Table 1:** Demographics and Clinical baseline characteristics

Clinical characteristics	Number of patients (%)
Age (Years)	**47 ± 11**
Male	22 (88)
Current Smoker	12 (48)
HBP	13 (52)
Dyslipidaemia	8 (32)
Diabetes mellitus	2 (8)
Family history of cardiovascular disease	4 (16)
Known vascular disease	2 (8)
**- Past CABG**	1 (4%)
- **Past PCI + LEAD**	1 (4%)
BMI (kg/m^2^)	25 ± 5.3
**Delay between first symptoms and invasive exploration (months) /*N*** **= 24**^**a**^	3.5 ± 12.5
**Flank or Abdominal sharp pain /*N*** **= 23**^**a**^	20 (87)
Abnormal urine dipstick / *N* = 20^a^	13 (65)
**Creatinine level (**μmol**/L) / N = 24**^**a**^	88.5 ± 22.5
**Glomerular filtration rate (mL/min) / N = 24**^**a**^	97.2 ± 58.5
eGFR < 60 mL/min	4 (16)
**White blood cells (/mm**^**3**^**) / N = 24**^**a**^	11,900 ± 5400
LDH (UI/L) / *N* = 13^a^	886 ± 421
CRP (mg/L) / N = 23^a^	61 ± 123
Typical computed tomography scan	20 (80)
Bilateral renal involvement	3 (12)

*BMI* Body mass index, *CABG* Coronary artery Bypass Graft, *CRP* C-reactive protein, *GFR* Glomerular filtration rate (Cockcroft-Gault), *HBP* High blood pressure, *LDH* Lactate dehydrogenase, *LEAD* Lower Limb Arterial Disease

^a^Data were available for N patients out of 25

The delay of diagnosis of RI was short, within a few hours or days from symptoms onset, but could not be calculated in one patient, whose only symptom was high blood pressure. One patient was diagnosed RI only after a third episode. The onset of symptom was then considered as his last episode of flank pain.

Most patients (84%) had normal renal function (eGFR > 60 mL/min) at time of diagnosis.

The majority of patients (80%) had a typical enhanced computed tomography scan, i.e. a wedge-shaped perfusion defect. Regarding the 5 remaining patients, the CT-Scan suggested a pyelonephritis. This misdiagnosis was strengthened by elevated white blood cell count and CRP. For these patients, a second medical visit occurred because of persisting symptoms, and the diagnosis of RI was secondarily made by a reinterpretation of the first scan, a second CT-scan or a renal MRI.

### Standard paraclinical exploration

The main results are resumed in Table 2*.* Atrial fibrillation on ECG Holter monitoring and altered left ventricular function without apical thrombus were both found in only one patient. Lower extremity and supra aortic arterial doppler, although not systematically performed, were more cost-effective and showed arterial abnormalities in 32 and 24% of patients. Enhanced abdominal CT-scan helped to raise suspicion under the infarction’s mechanism in 40% of patients, suggesting arterial dissection (28%), renal arteries atherosclerosis (12%) and/or fibromuscular dysplasia (12%).

**Table 2:** Standard paraclinical exploration

Pathological findings	Number of patients (%)
**Sinus rhythm on 12-lead rest ECG**	**25 (100)**
**Atrial arrhythmia on Holter ECG / N = 24**^**a**^	1 (4)
**TTE / TOE abnormalities**	3 (12)
- **Foramen ovale**	2 (8)
- **LVEF < 50%**	1 (4)
**Lower limb arterial doppler abnormalities /*N*** **= 17**^**a**^	4 (23.5)
**Supra-aortic vessel doppler abnormalities /*N*** **= 19**^**a**^	4 (21)
**Renal arteries abnormalities on contrast enhanced CT**	10 (40)
- **Fibromuscular dysplasia**	3 (12)
- **Atheroma**	3 (12)
- **Dissection**	7 (28)

*CT* Computerized tomography, *ECG* Electrocardiogram, *LVEF* Left ventricular ejection fraction, *TOE* Transoesophageal echography, *TTE* Transthoracic echocardiography

^a^Data were available for N patients out of 25

### Angiography and IVUS

Results are resumed in Table 3*.*

**Table 3:** Angiography and IVUS

Parameters and findings	Number of patients (%)
**Contrast agent volume (mL)**	**107 ± 43**
Renal artery angiography	
**Normal renal artery wall**	12 (48)
**Atherosclerosis without significant narrowing**	4 (16)
**Atherosclerosis with significant narrowing**	2 (8)
**Arterial dissection**	7 (28)
**Angiodysplasia**	2 (8)
**Arterial occlusion**	4 (16)
Renal artery IVUS /N = 23 ^a^	
**Normal renal artery wall**	11 (47.8)
**Atherosclerosis without significant narrowing**	5 (21.7)
**Atherosclerosis with significant narrowing**	2 (8.6)
**Renal artery dissection**	8 (34.8)
**Arterial wall hematoma**	1 (4.3)
**Complications of invasive explorations**	1 (4.3)

*IVUS* intravascular ultrasound

^a^Data were available for N patients out of 25

Invasive explorations were performed after a median time of 3.5 months from symptoms onset. Longer delays were found in patients referred from peripheral hospitals.

**Fig. 1:**
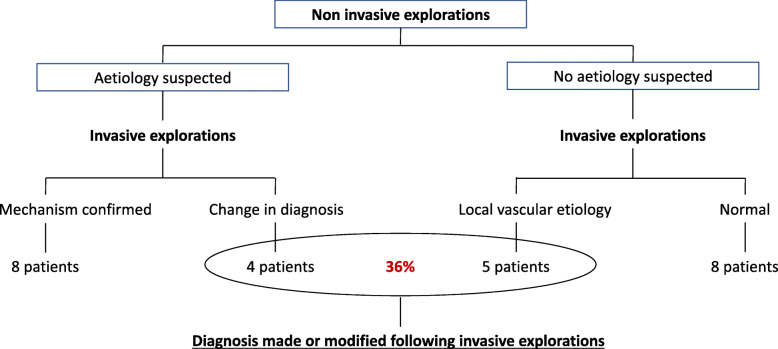
Impact of invasive explorations to confirm/infirm the diagnosis suspected following non-invasive investigations

Invasive exploration of renal arteries led to a change in the etiological category of RI, i.e. local vascular versus cardioembolic, in 9 patients (36%, see Fig. 1). In all patients where a local vascular mechanism was suspected following the abdominal CT-scan and confirmed by invasive investigations, the latter provided thorough details regarding the arterial wall, finely differentiating dissection from athero-thrombosis and fibromuscular dysplasia.

Only one complication occurred due to these invasive explorations: an arterial dissection due to IVUS guide wire introduction into a renal artery. This complication was immediately treated by angioplasty and stent implantation, followed by a dual antiplatelet therapy for 3 months, without any reported long-term related outcome, with a follow-up duration of 58 months.

**Fig. 2:**
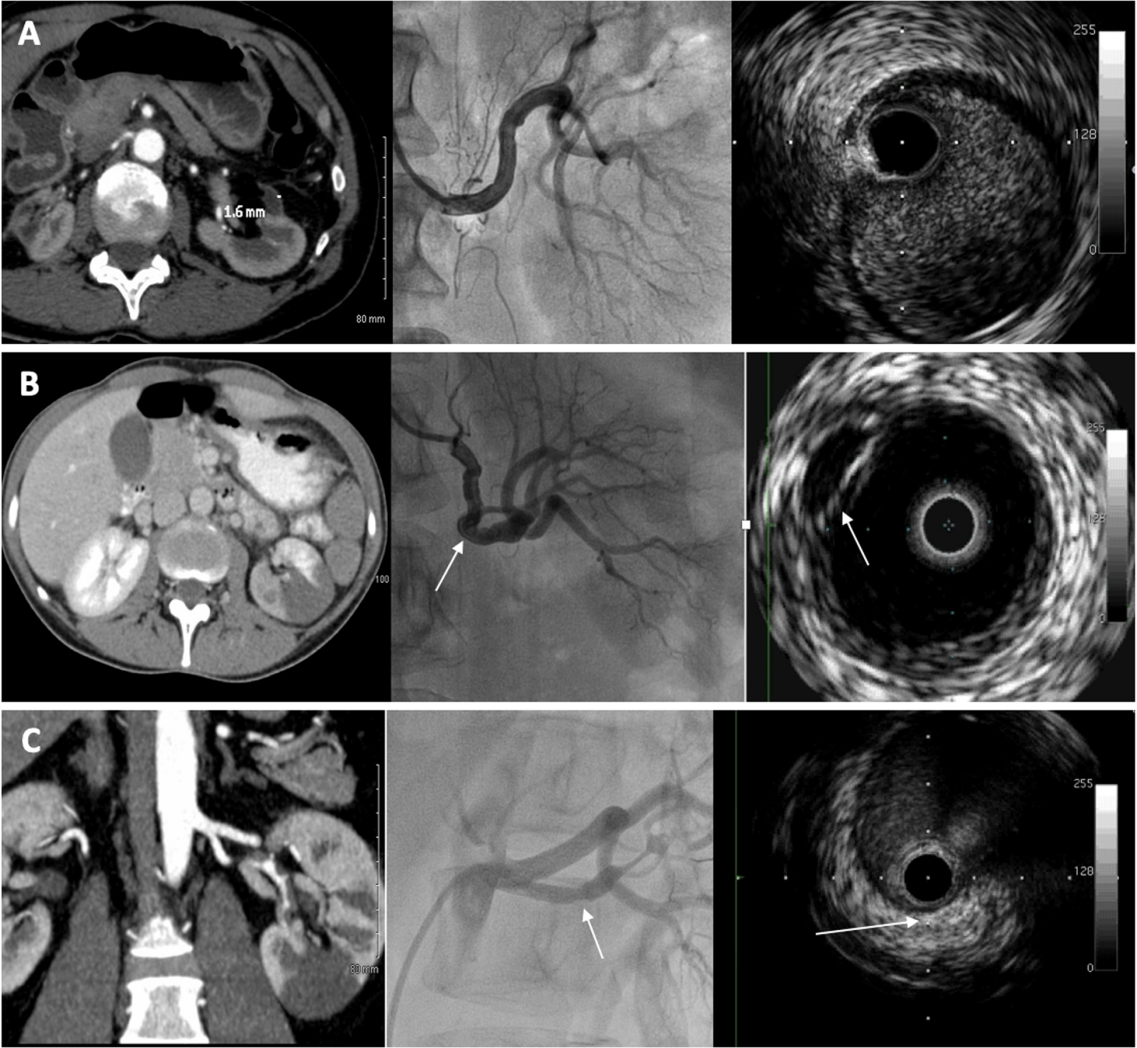
Examples of typical images in renal infarction patients. For each panel, the left image is a CT scan image (or reconstruction), the middle is the renal arteriography and the right image is an IVUS image. Panel **a** shows images from a patient with left renal infarction and no anomalies detected during the invasive exploration. Panel **b** shows images from a patient with left renal infarction and a spontaneous dissection visible on both renal angiography and IVUS (white arrow). Panel **c** shows images from a patient with left renal infarction and at least one calcified atherosclerotic plaque visible on both renal angiography and IVUS (white arrow)

IVUS could not be performed in one patient because of failure to insert the IVUS probe in both renal arteries. For another patient with arterial dissection visualized on angiography, the guide wire could not be inserted in the vessel’s true lumen. Figure 2 presents some typical images that were encountered in arteriography and IVUS.

Of note, in our cohort, no patient had normal renal angiography and a pathological IVUS exploration.

### Secondary prevention

Before invasive exploration, patients were mainly treated with oral or parenteral anticoagulation therapy (40%), or antiplatelet agents (36%). 16% were free of antithrombotic treatment. Few patients received a combination of anticoagulation and antiplatelet therapy (8%).

Invasive exploration led to a change in anti-thrombotic strategy in 56% of cases, with an increased prescription of antiplatelet therapy (72% at discharge versus 36% at admission) and a decreased use of anticoagulant therapy (40% at discharge versus 48% at admission). Few patients with a genuine indication for oral anticoagulant therapy were also diagnosed with renal atherosclerosis and were then treated with a combination of oral anticoagulant and antiplatelet therapy. Data are summarized in Table 4.

**Table 4:** Secondary prevention treatment

**Antithrombotic treatment at admission:**	**Number of patients (%)**
- None	4 (16)
- Anticoagulation therapy alone	10 (40)
- Antiplatelet therapy alone	9 (36)
- Both anticoagulation + antiplatelet therapies	2 (8)
**Antithrombotic treatment at discharge:**	
- Anticoagulation therapy	7 (28)
- Antiplatelet therapy alone	15 (60)
- Both anticoagulation + antiplatelet therapies	3 (12)
Change in anti-thrombotic strategy:	**14 (56)**

### Outcomes

Results are resumed in Table 5*.* No patient died during a median follow-up time of 59 months. No major adverse cardiovascular and cerebrovascular event was reported, although one patient was admitted for acute limb ischemia caused by apical thrombus.

**Table 5:** Outcomes

**Follow up time (Months)**	**59 ± 36**
**Current treatment**	**Number of patients (%)**
- None	3 (12)
- Anticoagulation therapy	3 (12)
- Antiplatelet therapy alone	17 (68)
- Both anticoagulation + antiplatelet therapies	2 (8)
Treatment change or discontinuation	8 (32)
**MACCE**	0 (0)
**Death**	0 (0)
**Renal infarction recurrence**	0 (0)
**Bleeding**	
- **BARC 0**	13 (52)
- **BARC 1**	9 (36)
- **BARC 2**	2 (8)
**Creatinine level (μmol/L) /*N*** **= 18**^**a**^	93 ± 16
**Renal insufficiency (eGFR < 60 mL/min)**	2 (8)
**End stage renal disease**	0 (0)

*BARC* Bleeding academy research consortium, *MACCE* Major adverse cardiovascular and cerebrovascular event

^a^Data were available for N patients out of 25

32% of patients had an antithrombotic treatment modification compared to the antithrombotic strategy prescribed at discharge. Two patients under antiplatelet and anticoagulant therapy were advised to discontinue anticoagulant by their nephrologist. Anticoagulant therapy was introduced in another patient after diagnosis of a left ventricular apical thrombus. One patient stopped antithrombotic treatment because of non-severe bleeding, and no severe bleeding was reported in the study. Hematemesis occurred in one patient, caused by mycotic gastritis under antiplatelet therapy.

Regarding renal function, among the 4 patients (16%) with eGFR < 60 mL/min at the time of invasive exploration, only 2 of them had persistent renal insufficiency. No end-stage renal disease with dialysis occurred during the follow-up.

## Discussion

Our study reports for the first time that invasive investigations of renal infarction, including renal arteriography and endovascular ultrasound, modified the secondary prevention treatment prescription through a better assessment of the aetiology of RI, leading to an increased prescription of antiplatelet treatment and a decreased use of oral anticoagulation.

### Secondary prevention

There was a change in RI secondary prevention treatment these last two decades. The systematic empirical anticoagulant treatment with vitamin-K antagonist was replaced by a case-by-case antithrombotic treatment, including anticoagulant and/or antiplatelet therapy, guided by a multidisciplinary etiological assessment [8]. This appears logical, as RI may be caused by numerous possible aetiologies that impact its presentation but also its outcome and risk or recurrence. Among these possible causes are atrial fibrillation, endocarditis, arterial dissection, lupus or sickle cell disease, fibromuscular dysplasia, sepsis, trauma, iatrogeny, the consequence of drug abuse and others [9]. In 2013, a classification of RI aetiologies into 4 groups was introduced by Bourgault et al. [10]: cardiac origin, associated with renal artery injury (vascular forms), associated with hypercoagulability disorders, and apparently idiopathic.

Concerning “vascular forms” of RI, Faucon et al. [11] stressed the importance of screening multiple vascular territories (carotid arteries, contralateral renal artery …) to strengthen the etiological investigations and decrease the rate of false idiopathic form. To our knowledge, renal artery invasive exploration, including IVUS, had never been evaluated in this purpose, further increasing the relevance of our proof-of-concept study.

In our study, after standard and invasive exploration, most patients were discharged with antiplatelet treatment alone (60%), versus 28% discharged with anticoagulant therapy and 12% discharged with a combination of antiplatelet and anticoagulant therapy. This is in clear contrast with the antithrombotic prescription immediately after the diagnosis of RI reported in the literature, that consisted mainly of oral anticoagulation (Table 6).

**Table 6:** Cohort studies of patients with renal infarction

***Study (number of patients) Period of inclusion***	***Mean age (Years)***	***History of atrial fibrillation (%)***	***ESRD***	***All-cause mortality rate***	***Recurrence of thromboembolic event***	***Antithrombotic secondary prevention treatment***		
						*anticoagulant alone*	*antiplatelet alone*	*anticoagulant and antiplatelet*
***Bourgault*et al.** [10] ***1989–2011***	*53*	*18*	*5% at 30 days*	*0% at 30 days*	*–*	*38*	*35*	*0*
***Huang*et al.** [3] ***1991–2016***	*56*	*56*	*7%*^*b*^*at 48 months*	*30% at 48 months*	*12% at 48 months*	*44*	*0*	*0*
***Oh*et al. **[15] ***1993–2013***	*60*	*45*	*2,1% at 20 months*	*5% at 20 months*	*2,8*	*48,2*	*37,2*	*14*
***Bae*et al.** [12] ***1995–2012***	*59*	*40*	*–*	*–*	*–*	*70*	*6*	*0*
***Mesiano*et al.** [13] ***1999–2015***	*59,8*	*28*	*5.6% at 15 months*	*–*	*–*	*44*	*33*	*0*
***Rhee*et al.** [1] ***2000–2009***	*56*	*25*	*–*	*19,7% at 40 months*	*–*	*–*	*–*	*–*
***Faucon*et al.** [11] ***2000–2015***	*53*	*4*	*–*	*–*	*–*	*–*	*–*	*–*
***Yun*** [2] ***2006–2012***	*61,3*	*53*	*2% at 41 months*	*36% at 5 years*^*a*^	*19% at 20 months*	*100 (for 3–6 months and lifelong if indication)*	*0*	*0*
Cerba et al. [14] ***2013–2015***	*57*	*–*	*0% at 6 months*	*9% at 6 months*	*0 at 6 months*	*64*	*36*	*0,9*
***Eren*et al.** [16] ***2015–2018***	*53*	*30*	*3% at 14 months*	*5% at 14 months*	*–*	*41*	*0*	*–*

Studies written in **bold** were prospective

^a^dialysis-free survival at 5 years: 64%. ^b^: 5 patients on 70

*ESRD* End-stage renal disease

The more frequent use of antiplatelet therapy in our study was a direct consequence of invasive exploration, leading to a change in antithrombotic strategy in 56% of cases, towards antiplatelet therapy most of the time. In other studies, anticoagulant therapy was more widely used because of a higher rate of atrial fibrillation (18 to 56% in the literature [1–3, 10, 12–16] versus 4% in our population), and probably because of a restrained use of antiplatelet therapy, some teams even considering that antiplatelet therapy has no place in the treatment of RI [2].

The mortality and recurrence of thromboembolic event rates were also very low in our population, in comparison with other studies (0% at 59 months versus at least 5% at 20 months respectively). This may be attributable to the secondary prevention treatment, yet some bias may partly explain these differences in outcome rates: our population included patients that were younger, therefore with fewer comorbidities, and without any clear evidence for a cardioembolic origin at the time of RI diagnosis, as shown by the lower rate of atrial fibrillation (4% versus 25–56%). This may therefore explain this difference in prognosis.

There were no hypercoagulability disorders diagnosed in our patients. Patients were only referred to a haemostasis specialist in case of normal standard and invasive exploration, or in case of haemostasis disorder on routine blood tests. This multidisciplinary approach was in agreement with previously published data to avoid futile biological assays [17].

### Safety

The safety of IVUS is well established, particularly in coronary artery disease. The complication rate varies from 0.5% up to 3% [18]. Long-term safety has also been evaluated, and coronary IVUS was not associated with a worsening of atherosclerosis at 24 months, compared with angiography alone [19]. Only one complication occurred in our study due to IVUS and not selective renal angiography and was successfully treated at the time of the exploration. Yet this pleads for a carefully thought use of IVUS. We propose that IVUS may be used in addition to renal angiography only when the latter was considered normal, to exclude the presence of a local vascular disorder, or inconclusive.

### Delay

In our cohort, no invasive exploration of RI was performed at the early phase. The diagnosis of RI is indeed often delayed, due to an unspecific clinical and biological presentation [20]. Moreover, patients were referred by the nephrology teams, to whom local centres had themselves often referred the patients, delaying the exploration all the more. However, following the same logic as in ST-segment elevation myocardial infarction, early renal artery angiography could allow percutaneous renal revascularization with in situ thrombolysis and/or angioplasty [21–23]. In case of massive RI with percutaneous treatment failure, surgical embolectomy or bypass graft may be an option [24].

Given the high risk of recurrence, even late diagnosed RI should undergo etiological exploration the sooner, in order to choose the most appropriate secondary prevention treatment as soon as possible.

### Renal function

4 patients (16%) had renal insufficiency at time of diagnosis (eGFR < 60 mL/min) and only 2 of them (8%) had persistent impaired renal function at the time of our follow-up. No end-stage renal disease was reported. This was consistent with the literature as all retrospective studies agree on RI’s rare evolution to renal insufficiency and dialysis [2, 3, 12].

Last, this was a retrospective study conducted on a limited number of patients and this is the major limitation of this work. Yet, despite the rarity of this pathology, its general bad prognosis plead for a better identification of its etiological mechanism and the present study offers new perspective of discussion.

## Conclusion

Our study is the first to suggest the usefulness of renal artery invasive exploration to complete the etiological investigation in patients with RI. This strategy, including renal artery angiography with/without IVUS, appears to be safe, improves the diagnosis process and efficiently guides the secondary prevention treatment in order to reduce thromboembolic recurrence, which is the main cause of mortality after RI. Considering the rarity of this disease, multicentric randomized studies are necessary to advocate the hypothesis that invasive exploration of renal artery can improve long-term prognosis.

## Data Availability

The datasets used and/or analysed during the current study are available from the corresponding author on reasonable request.

## Abbreviations

BARC: Bleeding Academic Research Consortium
CRP: C reactive protein
CT-scan: Computed tomography scanner
ECG: Electrocardiogram
GFR: Glomerular filtration rate
IVUS: Intravascular ultrasound
LDH: Lactate dehydrogenase
MACCE: Major Adverse Cardiovascular and Cerebrovascular Events
MDRD: Modification of Diet in Renal Disease
MRI: Magnetic resonance imaging
RI: Renal infarction
TOE: Transoesophageal echocardiography
TTE: Transthoracic echocardiography
